# Dynamic organelle changes and autophagic processes in lily pollen germination

**DOI:** 10.1186/s40529-024-00410-6

**Published:** 2024-01-26

**Authors:** Chih-Chung Yen, Chia-Mei Hsu, Pei-Luen Jiang, Guang-Yuh Jauh

**Affiliations:** 1 Institute of Plant and Microbial Biology, Academia Sinica, 11529 Taipei, Taiwan, ROC; 2 Department of Biotechnology, National Formosa University, Huwei Township, Yulin County, Taiwan; 3 Molecular and Biological Agricultural Sciences, International Graduate Program, National Chung-Hsing University, Academia Sinica, Taipei, Taiwan; 4 Biotechnology Center, National Chung-Hsing University, Taichung, Taiwan

**Keywords:** Pollen germination, Organelle dynamics, Autophagy, Vacuole formation

## Abstract

Pollen germination is a crucial process in the life cycle of flowering plants, signifying the transition of quiescent pollen grains into active growth. This study delves into the dynamic changes within organelles and the pivotal role of autophagy during lily pollen germination. Initially, mature pollen grains harbor undifferentiated organelles, including amyloplasts, mitochondria, and the Golgi apparatus. However, germination unveils remarkable transformations, such as the redifferentiation of amyloplasts accompanied by starch granule accumulation. We investigate the self-sustained nature of amylogenesis during germination, shedding light on its association with osmotic pressure. Employing BODIPY 493/503 staining, we tracked lipid body distribution throughout pollen germination, both with or without autophagy inhibitors (3-MA, NEM). Typically, lipid bodies undergo polarized movement from pollen grains into elongating pollen tubes, a process crucial for directional growth. Inhibiting autophagy disrupted this essential lipid body redistribution, underscoring the interaction between autophagy and lipid body dynamics. Notably, the presence of tubular endoplasmic reticulum (ER)-like structures associated with developing amyloplasts and lipid bodies implies their participation in autophagy. Starch granules, lipid bodies, and membrane remnants observed within vacuoles further reinforce the involvement of autophagic processes. Among the autophagy inhibitors, particularly BFA, significantly impede germination and growth, thereby affecting Golgi morphology. Immunogold labeling substantiates the pivotal role of the ER in forming autophagosome-like compartments and protein localization. Our proposed speculative model of pollen germination encompasses proplastid differentiation and autophagosome formation. This study advances our understanding of organelle dynamics and autophagy during pollen germination, providing valuable insights into the realm of plant reproductive physiology.

**Supplementary Information:**

The online version contains supplementary material available at 10.1186/s40529-024-00410-6.

## Introduction

Pollen development within the anther represents a captivating biological phenomenon wherein intricate processes such as cell division, gene regulation, and cellular differentiation converge to facilitate successful pollination (Lord and Russell 2002; Marchant and Walbot, 2022; Palanivelu and Preuss. 2000). Carbohydrates and lipids are major energy reservoirs in mature pollen, essential components for germination and tube growth (Pacini and Viegi 1995). These reserves, dispersed across various cellular compartments, contribute to osmoregulation and plasma membrane stability and confer pollen vitality during critical dehydration, dispersal, and germination (Pacini and Viegi 1995).

Starch, a prominent polysaccharide stored in mature pollen grains of many crop plants, is essential for pollen germination and tube growth. Deficiency in starch accumulation leads to the blockage of pollen germination and pollen tube growth due to the shortage of energy and membrane material provision (Lee et al. 2022). Thus, the dynamic interplay between starch content and viability becomes evident through the profound influence of starch hydrolysis on pollen lifespan and emission timing (Pacini and Hesse 2002). Furthermore, plastids, which often serve as reservoirs for starch, play a multifaceted role as repositories of carbohydrates and contributors to pollen tube growth (Clement and Pacini, 2001). Additionally, pollen grains harbor an abundance of lipids, such as triacylglycerols in lipid droplets, which are likely required for pollen germination (Bashir et al. 2013; Ischebeck, 2016). This observation raised the intriguing hypothesis that proper pollen germination necessitates endogenous lipids.

Macroautophagy, or autophagy, substantially impacts plant growth, development, and response to various stresses (Signorelli et al. 2019). Under undisturbed conditions, autophagy is a fundamental mechanism to maintain cellular equilibrium by progressively degrading cytoplasmic constituents. The extensive investigation underscores that impaired autophagy hampers seed yield and accelerates the onset of premature leaf senescence (Barros et al. 2017; Chung et al. 2010; Phillips et al. 2008). Conversely, elevated autophagy levels stimulate growth, enhance seed yields, and facilitate remobilization (Chen et al. 2019a; Minina et al. 2018). Remarkably, autophagy induction occurs during both senescence processes and in response to stressors, effectively recycling cellular components (Su et al. 2020).

Despite these valuable insights, the intricate mechanisms orchestrating organelle differentiation and mobilization during pollen germination remain enigmatic. Drawing inspiration from extensively studied autophagy processes observed in yeast and mammalian systems, this study unveils processes analogous mechanisms underlie pollen germination (Taylor and Hepler 1997). By investigating ultrastructural changes during germination, encompassing organelle re-differentiation, the role of tubular ER in encapsulating amyloplasts and lipid bodies, and the intriguing presence of starch granules, lipid bodies, and membrane remnants within vacuoles, this investigation aims to elucidate potential autophagic processes involved in pollen development. Additionally, immunogold labeling sheds light on the specific protein localization within these processes, thereby advancing our understanding of the possible involvement of autophagic machinery in shaping organelle differentiation and vacuole formation. Through these explorations, this study not only enhances our comprehension of autophagy’s role in pollen development but also underscores its novel contributions of autophagy’s role in pollen development and its novel contributions to organelle dynamics and vacuole biogenesis. This study offers novel insights into cellular processes during germination and growth by unraveling the intricate relationship between autophagy and pollen development.

## Results

### Organelle dynamics during pollen germination

**Fig. 1:**
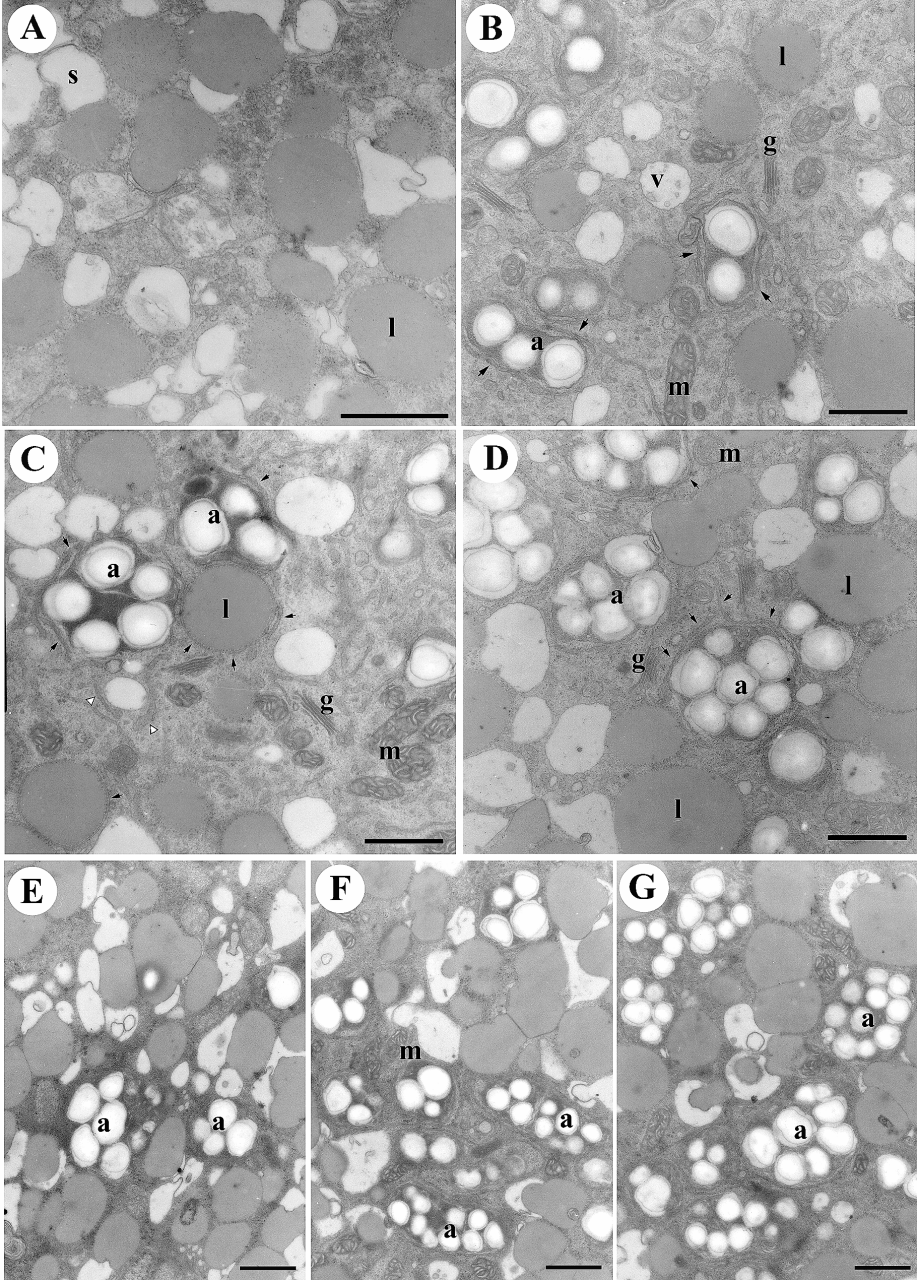
Dynamic amyloplast morphogenesis during pollen germination. Lily pollen grains underwent germination in a culture medium with either 290 mM sucrose (**A-D**) or 290 mM pentaerythritol (**E-G**) for varying time intervals: 0 min (**A**), 30 min (**B and E**), 60 min (**C and F**), and 90 min (**D and G**). The samples were rigorously fixed and subsequently embedded in Spurr’s resin to facilitate high-resolution ultrastructural analysis. In the initial stage, mature lily pollen grains exhibited minimal to no presence of starch granules or amyloplasts (**A**). However, as the process of pollen germination advanced, starch granules commenced accumulating within amyloplasts (**B-D**). Simultaneously, the quantity of starch granules increased proportionally with the expansion of amyloplasts. Notably, this pattern of amyloplast development persisted even when pollen grains were germinated without sucrose (**E-G**). In panels (**B-D**), solid arrows denote double-membrane structures enveloping differentiating amyloplasts, whereas open arrows indicate those enclosing lipid bodies. Key cellular components are appropriately labeled: a (amyloplast), g (Golgi apparatus), l (lipid body), m (mitochondria), v (vacuole). The Scale bars represent 1 μm, ensuring precise measurement in the ultrastructural analysis

Pollen germination is a pivotal event in the life cycle of flowering plants, signifying the transformation of quiescent pollen grains into actively growing structures. In our pursuit to unravel the intricacies of this process, we conducted a thorough investigation under precisely controlled culture conditions, with a primary focus on the ultrastructural alterations observed in germinating lily pollen grains at various time intervals. At the outset, desiccated mature pollen grains displayed organelles in a relatively undifferentiated state, comprising amyloplasts, mitochondria, and the Golgi apparatus (Fig. 1A). However, as the germination process advanced, a series of remarkable transformations unfolded. Subsequent to 30, 60, and 90 min of culture under sucrose treatment (Fig. 1B–D), our observations revealed distinct secretory vesicles emerging within the Golgi apparatus and the characteristic cristae of elongated mitochondria. The most noteworthy among these alterations was the redifferentiation of amyloplasts, originating from proplastids, and the subsequent accumulation of numerous starch granules (Fig. 1).

**Fig. 2:**
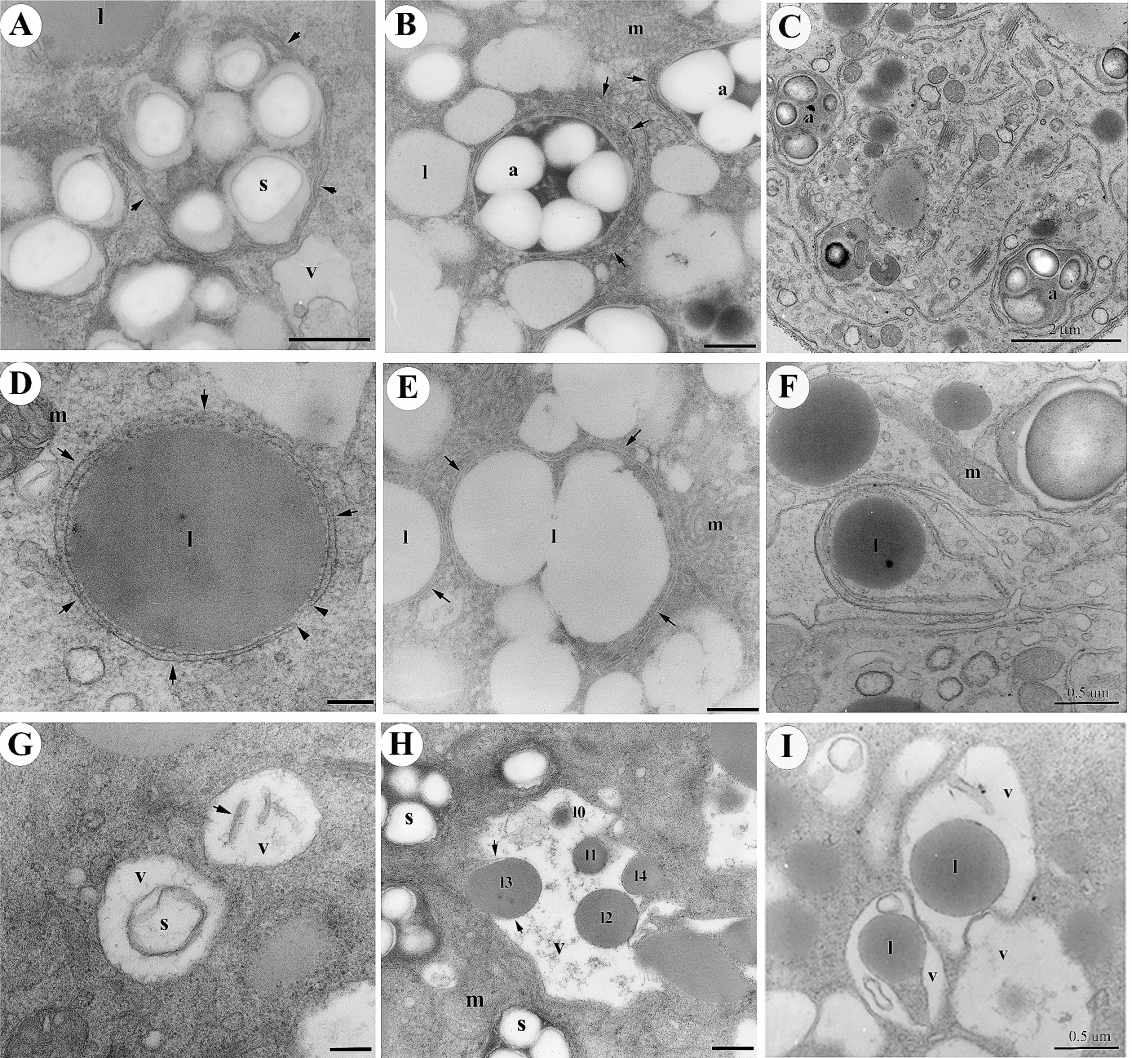
Autophagosome-like compartments enclosing amyloplasts and lipid bodies during lily pollen germination and tube elongation. Lily pollen grains, subjected to in vitro germination for 60 min (**A-B**; **D-E**; and **G-H**) or in vivo-grown pollen tubes collected from 2-day pollinated styles (**C–I**), were carefully embedded in Spurr’s resin (**A, C, D, F, G-H**) or prepared using rapid-freeze and freeze substitution techniques (**B, E**) for subsequent ultrastructural examination. A-B. Throughout lily pollen germination and pollen tube elongation process, tubular structures originating from the tubular endoplasmic reticulum (ER) double membrane structure, indicated by arrows, encased distinct organelles, specifically amyloplasts (**A-C**) and lipid bodies (**D-F**), culminating in the formation of autophagosome-like compartments. Sections of samples prepared using rapid freeze and freeze substitution techniques unveiled autophagosome-like structures encompassing amyloplast (**B**) and lipid bodies (**E**). Occasionally, partially digested starch granules (s) and membrane debris (arrow) were found in the vacuolar lumen (v, in G). (**H-I**) are typical vacuolar lumens containing various stages of engulfed lipid bodies, especially in H starting from on-the-way delivery into the vacuole (I3 and I4), engulfed into the vacuolar lumen (I1 and I2), and digested in the vacuole (I1), I0 signifies the eldest uptaken lipid body with only a small portion of the lipid body is visible within the vacuolar lumen. Significant cellular components are denoted as follows: a: (amyloplast), g (Golgi apparatus), l (lipid body), m (mitochondria), s (starch granule), v (vacuole). Scale bars = 1 μm (**A**), 0.2 μm (**B**), 0.1 μm (**C**), 0.5 μm (**D–I**)

### The role of osmotic pressure in amylogenesis

A fundamental question arousing concerning the impact of sucrose on amylogenesis in the culture medium. To investigate this, we introduced pentaerythritol, a slow-absorbing osmotic pressure modulator not metabolized by germinating lily pollen (Dickinson 1978). In this study, we treated the culture medium with 290 mM pentaerythritol to replicate the 290 mM sucrose osmotic condition. It’s important to note that pentaerythritol cannot serve as a source for carbohydrate metabolism during pollen development. Through an extensive comparative analysis of germination under sucrose-rich conditions (Fig. 1B-D) and pentaerythritol-supplemented media (Fig. 1E-G), a consistent finding emerged -the formation of amyloplasts persisted regardless of the medium composition. This intriguing discovery emphasized that the initiation of amylogenesis is not influenced by variations in osmotic pressure. Furthermore, a systematic exploration using pentaerythritol underscored the autonomous re-differentiation of amyloplasts during pollen germination, revealing a novel regulatory network in pollen development where starch biosynthesis and osmotic pressure are interconnected. Our results indicate that the formation of amyloplasts during lily pollen germination is a robust process independent of the medium’s osmotic conditions, highlighting the autonomous nature of amylogenesis and its potential regulatory links to starch biosynthesis and osmotic pressure.

### Ultrastructural insights into organelle changes and autophagy in lily pollen germination

In our study, we present significant findings that shed light on the involvement of autophagic machinery in lily pollen germination and pollen tube elongation. Our investigation encompassed both in vitro germination (2A-B, D-E, and G-H) and the examination of in vivo-grown pollen tubes collected from 2-day pollinated styles (2C, F, I). To gain thorough insights, we conducted an extensive ultrastructural examination using specimens embedded in Spurr’s resin (2A, C, D, F, G-H) or prepared with rapid freeze and freeze substitution techniques (2B, E). Our observation unveiled the pivotal role of tubular structures originating from the tubular endoplasmic reticulum (ER), indicated by arrows, in encapsulating distinct organelles, namely amyloplasts (2A-C) and lipid bodies (2D-F). These processes culminated in the formation of autophagosome-like compartments. Notably, our examination of specimens prepared using rapid freeze and freeze substitution techniques provided finer details of the autophagosome-like structures encompassing amyloplasts (2B) and lipid bodies (2E). Furthermore, we identified instances of partially digested starch granules (s) and membrane debris (arrow) within the vacuolar lumen (v, in 2G). Subsequent examination of typical vacuolar lumens (2H-I) demonstrated various stages of engulfed lipid bodies, especially in H, illustrating their journey from delivery into the vacuole (I3 and I4), engulfment into the vacuolar lumen (I1 and I2), and ultimate digestion within the vacuole (I1). These findings provide valuable insights into organelle dynamics during pollen germination and underscore the integral role of autophagic processes in shaping the fate of amyloplasts and lipid bodies during lily pollen development.

### Modulation of pollen germination and lipid body redistribution by autophagy inhibitors

**Fig. 3:**
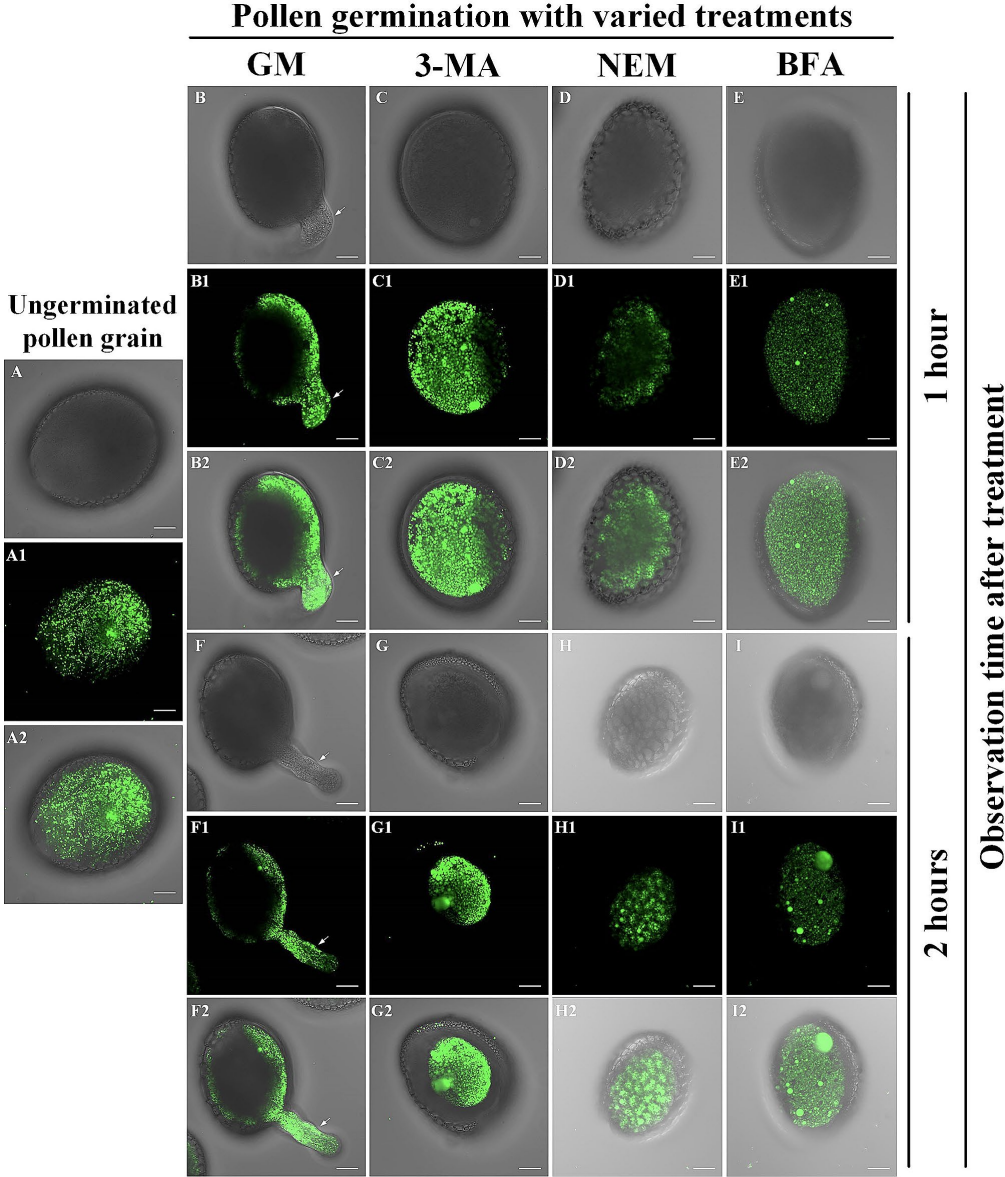
Impact of autophagy inhibitors on lipid body redistribution. Dynamic redistribution of pollen grain lipid bodies during 1–2 h of germination in the germination medium, as revealed by BODIPY 493/503 staining and Confocal fluorescence microscopy. The effects of pre-treatment with autophagy inhibitors on mature pollen germination are depicted. Arrows indicate the locations of pollen tubes. (**A**): Pollen grains before germination. (A1) Distribution of lipids within pollen observed using BODIPY 493/503 staining. (A2) Merged image illustrating lipid distribution within the cell. (**B**): Pollen grains after 1 h of germination in germination medium (GM). (**C, D, E**): Pollen grains pre-treated with 3-MA, NEM, and BFA, respectively, followed by 1 h of germination observation. (**F**): Untreated pollen grains germinated in a culture medium for 2 h. (**G, H, I**): Pollen grains pre-treated with 3-MA, NEM, and BFA, respectively, followed by 2 h of germination observation. Scale bar: 20 μm

In this thorough investigation, we focused on the effects of three autophagy inhibitors: 3-methyladenine (3-MA), N-ethylmaleimide (NEM), and Brefeldin A (BFA) on pollen germination and growth. Our examination revealed significant hindrances in the developmental process when these inhibitors were introduced. The application of 3-MA, NEM, and BFA resulted in pronounced disruptions in both pollen germination and subsequent growth, as illustrated in (**Figures** S1A and S1B). Of particular interest was the substantial impact of BFA on lily pollen germination. While germination remained feasible under its influence, it was accompanied by a severe impairment in pollen tube elongation (**Figure **S1C). Notably, 3-MA, employed at a concentration of 10mM, served as a potent autophagy inhibitor in our study. The choice of this concentration aligns with established practices in the field and is justified by the pivotal role of PI3K in autophagy activation. 3-MA is widely recognized as a PI3K inhibitor and is commonly employed to impede autophagic processes (Huang and Sinicrope 2010; Petiot et al. 2000; Seglen and Gordon 1982; Heckmann et al. 2013). This thorough analysis offers valuable insights into the complex interrelationship between autophagy and lily pollen development. Our investigation also delved into the dynamic redistribution of lipid bodies during pollen tube development and its modulation by autophagy inhibitors. Utilizing BODIPY 493/503 staining, we observed significant changes in lipid body distribution associated with pollen tube growth and polarization. In quiescent, non-germinating pollen grains, lipid bodies were uniformly dispersed (Fig. 3A). However, during the initial hour of pollen tube germination, a noticeable polarized growth pattern emerged as the pollen tube elongated from its tip (Fig. 3B). Lipids played a pivotal role by facilitating cellular membrane remodeling and promoting pollen tube elongation. Most notably, we observed the clustering of lipid bodies towards the polarized tip, emphasizing their essential contribution to directional pollen tube growth. Approximately two hours after initiation, as the pollen tube continued to to elongate, there was a marked reduction in lipid body quantity within the pollen grain, with most being nearly depleted (Fig. 3F). This suggests that the transfer and reorganization of lipid bodies within the pollen tube likely concluded by this stage, providing crucial resources for directional growth and reducing the overall lipid body count within the pollen cell.

To further explore the role of autophagy in this process, we subjected pollen grains to autophagy inhibitors, namely 3-MA, NEM, and BFA, followed by observation of lipid body dynamics using BODIPY 493/503 staining (Fig. 3). Remarkably, within the initial hour of treatment with these inhibitors, we did not observe the emergence of a polarized pattern in pollen tube germination, in contrast to untreated pollen grains (Fig. 3C, D and E). While normal, untreated pollen grains exhibited distinct lipid body movement towards the pollen tube after two hours of germination (Fig. 3F), this phenomenon was notably absent in pollen grains treated with the autophagy inhibitors 3-MA, NEM, and BFA (Fig. 3G, H and I).

### Effects of BFA on organelles and cellular processes

**Fig. 4:**
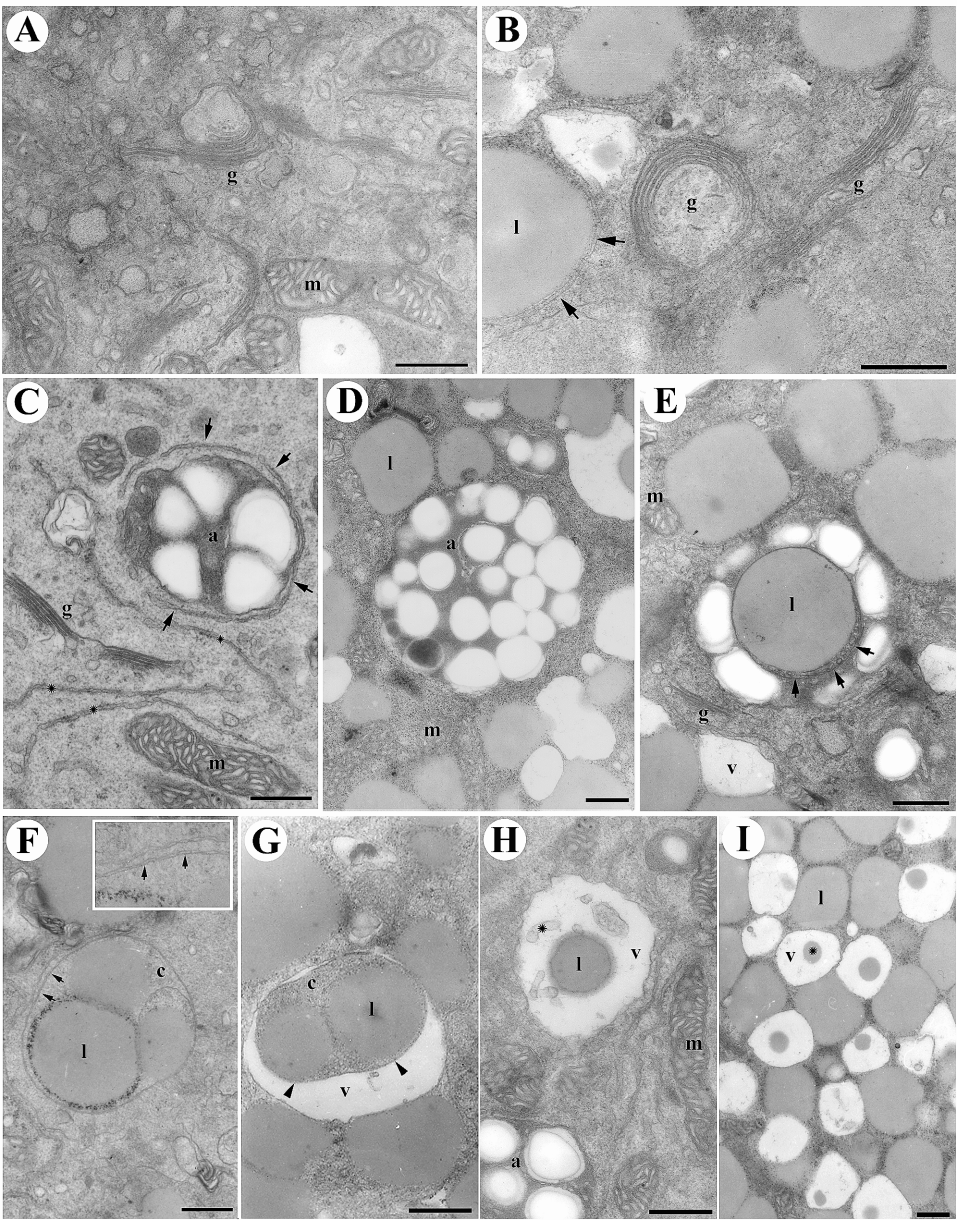
Effects of BFA on lily pollen ultrastructure. Lily pollen grains were germinated in a medium with 1 µg/ml BFA for 1 h and analyzed using ultrastructural analysis. (**A-B**). BFA led to significant changes in the Golgi apparatus, with elongated cisternae forming concentric circles and partially enclosing cytoplasm. (**C**): Amyloplasts developed normally with an inner membrane system and abundant starch granules and tubular ER began enclosing these amyloplasts (indicated by arrows). (**D**): Amyloplasts contained starch granules and, occasionally, lipid bodies enclosed by double membranes (arrows). (**E**): Unveils a distinctive ultrastructural characteristic observed during the germination of lily pollen in the presence of 1 µg/ml BFA for 1 h. The picture emphasizes the envelopment of lipid bodies by double-membrane structures (as indicated by arrows). (**F**): Double-membrane structures enclosed lipid body clusters and cytoplasm in the vegetative cytoplasm. The inner image shows the double membrane’s characteristics (arrows). (**G-H**): Membrane-enclosed contents, including lipid bodies and cytoplasm resembling inclusion bodies, were transported to the vacuole (**G**). Inside the vacuole, the lipid body clusters and inner membrane gradually underwent digestion, leaving behind degraded membrane debris and lipid bodies (**H**). Occasionally, some lipid bodies remained in the vacuoles (**I**). Key components: a: amyloplast, g: Golgi apparatus, l: lipid body, m: mitochondria, s: starch granule, v: vacuole. Bars = 1 μm (**A, B**), 0.5 μm (**C-I**)

A thorough investigation of lily pollen grains exposed to BFA for an hour unveiled intriguing effects on the Golgi apparatus, mitochondria, amyloplast differentiation and the fate of lipid bodies. BFA treatment induced significant alterations in the Golgi apparatus’s morphology and structure, where the cisternae extended and stacked as concentric circles, engulfing a portion of the cytoplasm (Fig. 4A and B). This transformation is a known consequence of BFA treatment, disrupting vesicular transport and protein secretion (Klausner et al. 1992). Notably, mitochondria remained largely unaffected by BFA treatment, maintaining their characteristic shape and cristae, indicative of normal functionality (Fig. 4A and C). In the presence of BFA, amyloplasts exhibited distinctive characteristics, including a well-developed inner membrane system and an abundance of starch granules enclosed within tubular ER structures (Fig. 4C). Coexistence of excessive starch granules and lipid bodies within amyloplasts underscored the complex processes influenced by BFA. Furthermore, lipid bodies were observed, highlighting the complex processes influenced by BFA(Fig. 4D and E). Lipid bodies were frequently observed enclosed within a double-layered membrane, signifying their entrapment within autophagosome-like structures (Fig. 4F). Furthermore, membrane-enclosed content, including lipid bodies and a portion of cytoplasm with a morphology resembling and inclusion body, was delivered to the vacuole. Within the vacuole, the enclosed lipid body clusters and their inner membranes underwent gradual digestion, leaving behind degraded membrane debris and/ or lipid bodies. Occasionally, remnants of lipid bodies were found within the vacuoles. These results collectively suggest that BFA-induced autophagy disruption plays a critical role in pollen germination and organelle dynamics, emphasizing the intricate interplay between autophagy and pollen development.

### ER involvement in autophagosome-like compartment formation

**Fig. 5:**
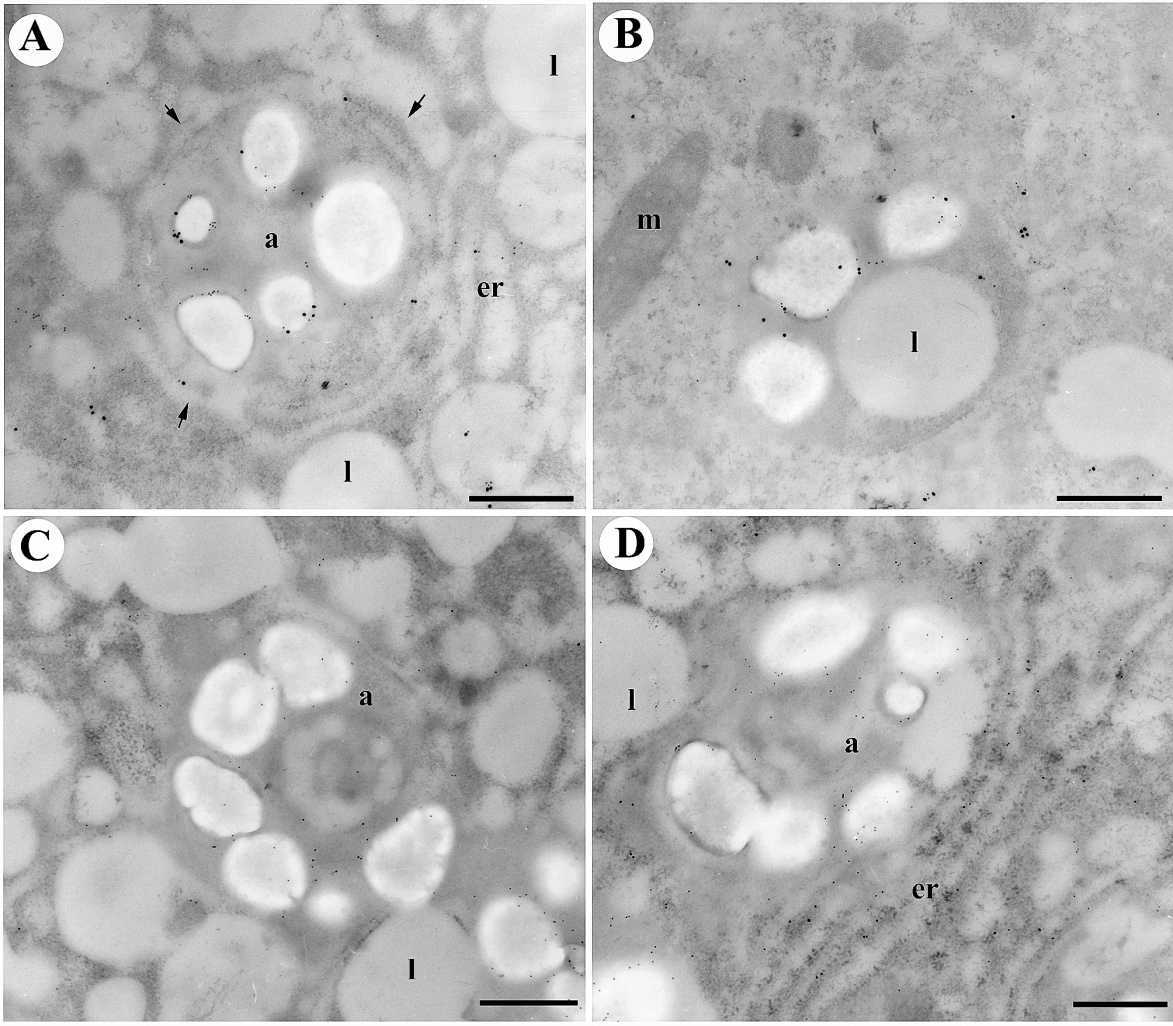
Co-localization of Wx protein, BiP, α-amylase, and SH-EP in amyloplasts of germinating lily pollen. Desiccated lily pollen grains, subjected to 60 min of germination in a culture medium, were embedded in LR White resin for subsequent immunocytochemical examination utilizing specified antibodies. (**A-B**): Immunogold localization revealed that the Wx protein (10 nm gold particles) and the endoplasmic reticulum (ER)-resident protein BiP (18 nm gold particles) exhibited specific localization on the periphery of starch granules and within the stromal regions of amyloplasts. Notably, neither protein was detected inside the starch granules (**A**) or within the enclosed lipid bodies (**B**). BiP was also identified in the tubular ER surrounding the amyloplasts (indicated by arrows in A). (**C-D**): Immunogold labeling of α-amylase (10 nm gold particles in C) and SH-EP (10 nm gold particles in D) demonstrated their specific and predominant localization on the margins of starch granules and within the amyloplast stroma. Moreover, SH-EP proteins were observed in the ER and the tubular ER enveloping the amyloplasts (arrows in D), while being absent from lipid bodies (**D**) and the cytoplasm. Legend Key: a: amyloplast, er: endoplasmic reticulum, l: lipid body, m: mitochondria. Scale Bar: 0.5 μm

To affirm the membranous nature of the ER and its involvement in the formation of autophagosome-like compartments, we conducted immunogold labeling experiments targeting specific proteins during amyloplast differentiation in lily pollen germination. The immunogold labeling of Waxy (Wx, a starch granule localized protein) localized to starch granules and binding immunoglobulin protein (BiP, an ER lumen localized protein), revealed their predominantly localized to the peripheries of starch granules and within the amyloplast matrix **(**Fig. 5A and B**)** (Li et al. 2020; Pobre et al. 2019). This co-localization suggests that the ER plays a significant role in the formation of autophagosome-like compartments. The proximity of these proteins to the starch granules and amyloplast matrix indicates their involvement in the dynamics of organelles during pollen germination. Immunogold labeling of α-amylase, an enzyme found in starch granules in amyloplasts, and sulfhydryl-endopeptidase (SH-EP) a vacuole localized protein, exhibited substantial labeling on starch granules and within the amyloplast matrix **(**Fig. 5C and D**)**. Additionally, the presence of SH-EP proteins within the ER and the envelopment of tubular ER structures around amyloplasts confirmed that these autophagosome-like compartments originate from the ER. This co-localization of proteins with starch granules and within the amyloplast matrix further supports the involvement of the ER in organelle dynamics. The immunogold labeling experiments provided strong evidence for the role of the ER in the formation of autophagosome-like compartments and organelle dynamics during lily pollen germination. These findings enhance our understanding of the complex processes involved in plant reproductive physiology.

### Subcellular localization of ATG8 in germinating lily Pollen

**Fig. 6:**
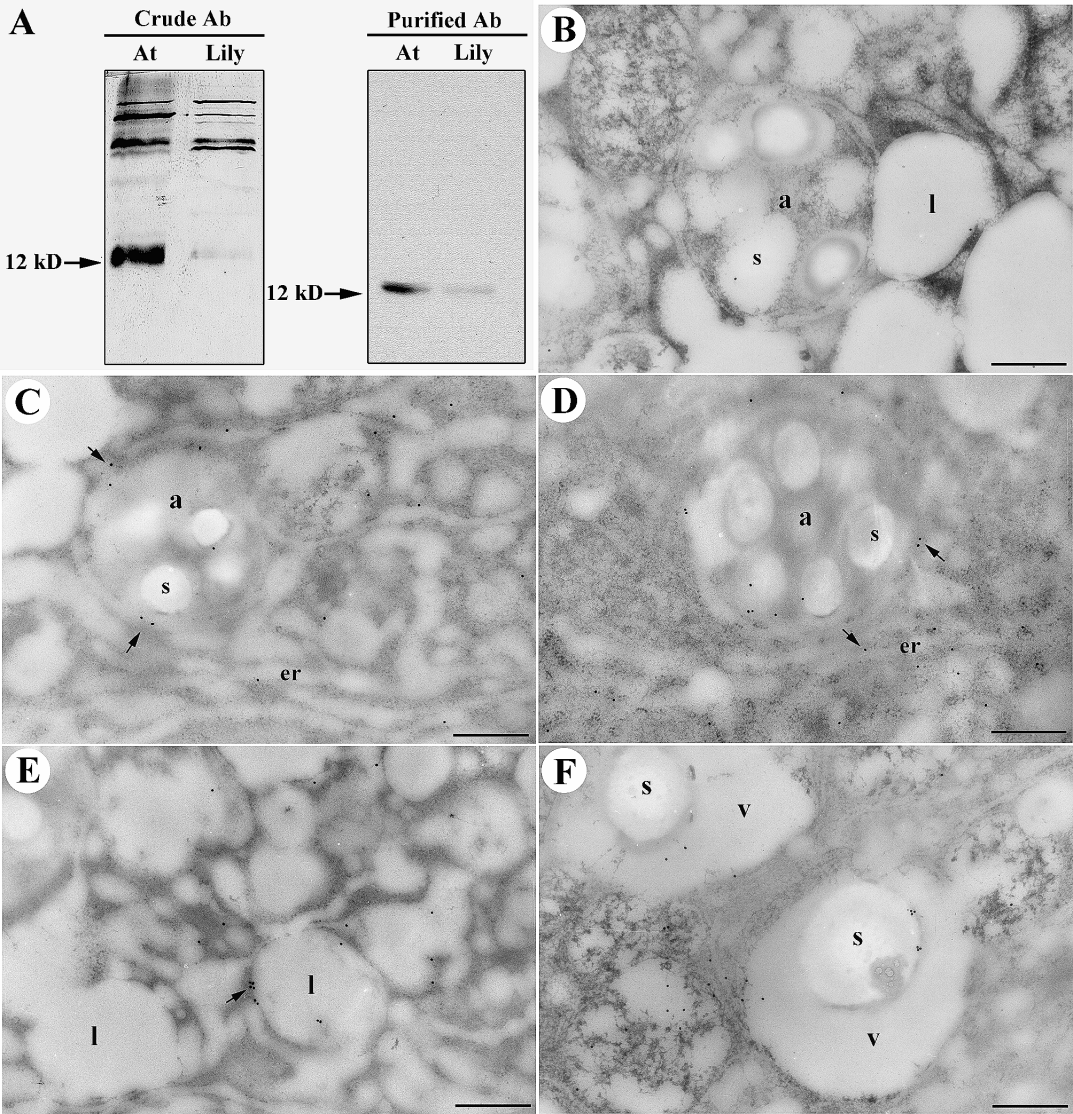
Presence and subcellular localization of ATG-8 in germinating lily pollen. (**A**): Total proteins extracted from *Arabidopsis* seedling (At) or 60-minute germinating lily pollen (Lily) were subjected to immunoblotting analysis using anti-ATG8 antiserum, revealing multiple recognized bands by the anti-ATG8 antiserum (left panel). Immunoblotting analysis with affinity-purified anti-ATG8 antiserum identified a single major ATG8 protein in *Arabidopsis* seedlings and germination lily pollen (right panel in A). Desiccated lily pollen grains, germinated for 60 min in a culture medium, were embedded in LR White resin for subsequent immunocytochemical examination employing control (secondary antibody only, B) and affinity-purified anti-ATG8 antiserum (**C-D**). Only a minimal number of gold particles were recognized by the secondary antibody (**B**). Numerous ATG8 orthologous proteins were detected in tubular ER structures surrounding amyloplasts (arrows in C-D), oil bodies (arrow in **E**), and the degraded amyloplast/ starch granules (s) within the vacuolar lumen (v, in **F**). Legend Key: a: amyloplast, er: endoplasmic reticulum, l: lipid body, m: mitochondria, S: starch. Scale Bar: 0.5 μm

In order to elucidate the subcellular localization and expression of ATG8 in germinating lily pollen, we employed immunogold labeling. Firstly, we conducted immunoblotting analysis to assess the presence of ATG8 in Arabidopsis seedlings (At) and germinating lily pollen (Lily). The left panel of Fig. 6 shows multiple bands recognized by anti-antiserum, indicating the presence of ATG8 in both samples. The right panel of Fig. 6 illustrates the results obtained with affinity-purified anti-ATG8 antiserum, which revealed a single major ATG8 protein in Arabidopsis seedling and germinating lily pollen (Fig. 6A). Subsequently, desiccated lily pollen grains subjected to 60 min of germination were embedded in LR White resin for immunocytochemical examination. We performed immunolabeling experiments using a control (secondary antibody only, B) and affinity-purified anti-ATG8 antiserum (Fig. 6C and D). Few gold particles were observed in the control group (Fig. 6B). In contrast, numerous ATG8 orthologous proteins were detected within the tubular ER structures surrounding amyloplasts (Fig. 6C and D), as well as within lipid bodies (Fig. 6E), the degraded amyloplast/ starch granules (s), and the vacuolar lumen (v). These findings collectively provide compelling evidence that ATG8 homologous proteins primarily associate with subcellular structures intricately related to autophagy, including amyloplasts, lipid bodies, and vacuoles.

## A hypothetical model of pollen germination: three distinct stages of autophagic processes

**Fig. 7:**
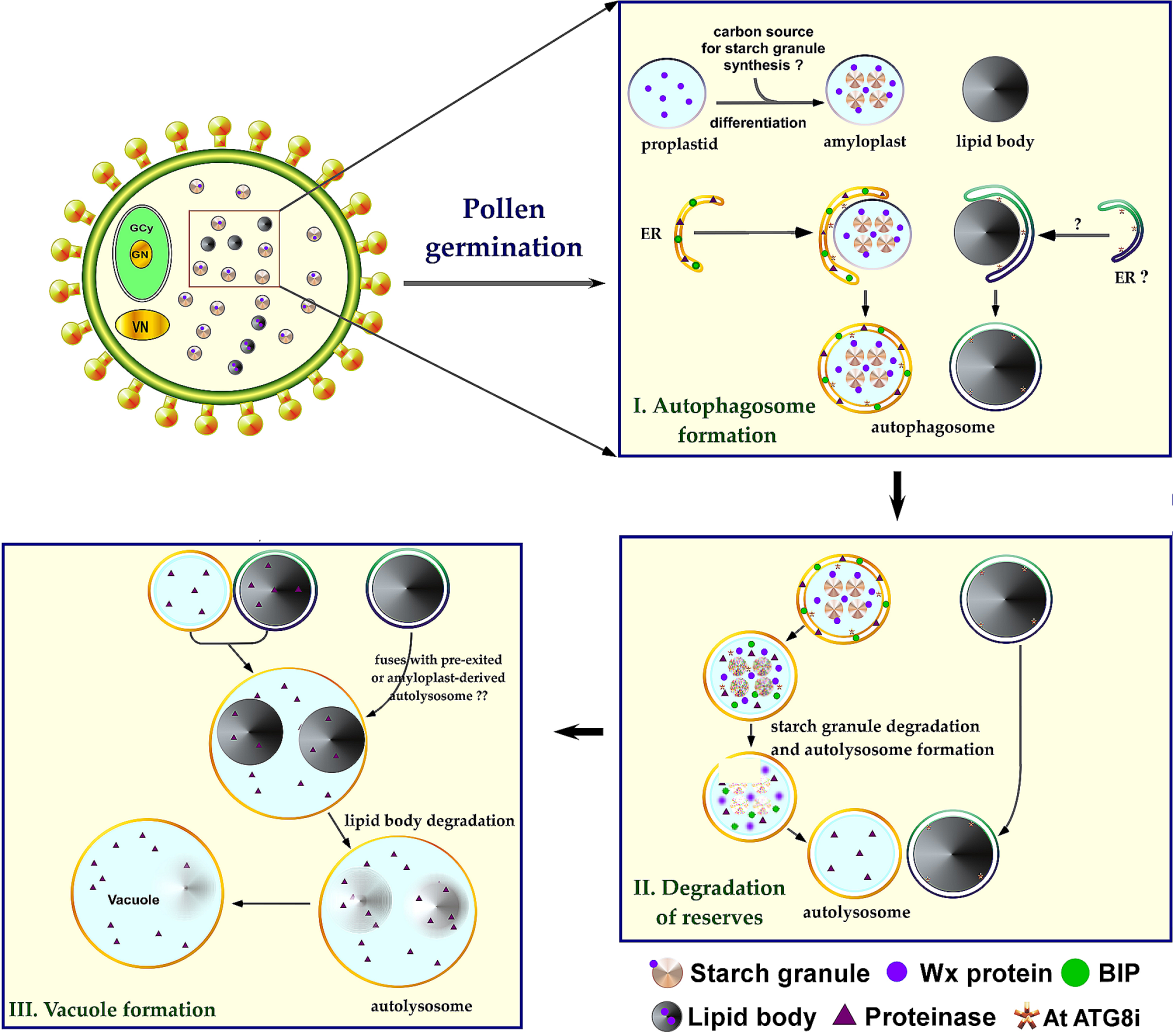
Proposed working hypothesis for the dual autophagic mechanisms involved in reserved material transport and vacuole ontogeny during pollen germination.This schematic representation outlines the hypothesized dual autophagic pathways involved in the transportation of stored materials and the development of vacuoles during the progression of pollen germination

During pollen germination, the proplastid in the pollen grain transforms into an amyloplast, producing starch granules from carbon sources. This process is closely tied to the endoplasmic reticulum (ER), ultimately leading to the creation of autophagosomes (He et al. 2023; Hikita et al. 2018; Zheng et al. 2019). Autophagosomes are responsible for the breaking down of amyloplasts in the columella cells of Arabidopsis roots (Nakayama et al. 2012). This differentiation process is intricately connected to the ER, culminating in autophagosome formation. Additionally, autophagosome-like structures envelop lipid bodies (Zienkiewicz and Zienkiewicz 2020), resulting in the creation of a distinctive double-membrane structure recognized as an autophagosome- a critical step known as autophagosome formation (Fig. 7).

Subsequently, the stored reserves undergo degradation during the following phase of pollen grain germination. This complex process involves the breakdown of starch granules and lipid bodies, orchestrated through the formation of autolysosomes. Transitioning the third stage, a fusion event occurs between lipid bodies and autolysosomes. In this fusion process, lipid bodies merge with pre-existing autolysosomes or those derived from amyloplasts. Remarkably, the lipid bodies within the autolysosomes degrade after this fusion event. The degradation of lipid bodies within the autolysosomes plays a pivotal role in vacuole formation, marking a critical juncture where the pollen grain initiates the growth of the pollen tube. This phase holds exceptional significance, emphasizing the shift from reserve utilization to active pollen tube growth (Fig. 7).

## Discussion

The interplay relationship between autophagy and lily pollen germination holds profound significant in the realm of plant developmental biology. Autophagy, the cellular process responsible for recycling and eliminating various cellular components, plays a pivotal role in the transition from quiescent pollen germination to active growth phases (Wang and Liu 2013; Wada et al., 2008), providing valuable insights into this complex process. During pollen germination, autophagy efficiently eliminates redundant organelles, including mitochondria, chloroplasts, lipid bodies, and starch granules, signifying its integral involvement in this intricate process.

An intriguing hypothesis arises regarding a mechanism reminiscent of mitochondrial translocation, enabling the entry of membranous structures into amyloplasts during pollen germination (Abeliovich et al. 2000; Smith-Huerta 1996). Close examination at the ultrastructural level consistently reveals the envelopment of developing amyloplasts and lipid bodies by the tubular ER. This observation suggests the activation of autophagic pathways akin to those observed in yeast under nutrient-depleted conditions.

The inhibiting autophagy profoundly affects the dynamics of lily pollen germination. Disrupting the autophagic machinery significantly impedes fundamental aspects of pollen development and growth (Chen et al. 2019b). Autophagy inhibitors exert selective effects on pollen tube elongation, underscoring the multi-dimensional involvement of autophagy across various germination phases (Chung et al. 2010). Notably, autophagy inhibition disrupts the differentiation of crucial organelles such as amyloplasts and results in the accumulation of structures resembling lipid bodies, indicative of compromised cellular component recycling. The use of autophagy inhibitors, including 3-MA, and NEM unequivocally underscores the indispensable role of autophagy in steering successful germination **Figure **S1). The effects of BFA treatment on lily pollen grains reveal significant alterations in various organelles and cellular processes, shedding light on the intricate interplay between autophagy and pollen development (Fig. 3; S1) (Šamaj et al. 2004). BFA-induced changes in the Golgi apparatus are consistent with previous findings, where BFA disrupts vesicular transport and protein secretion. The elongation and concentric stacking of Golgi cisternae indicate the interference of BFA with normal cellular processes (Fig. 4). This alteration in Golgi morphology highlights the crucial role of vesicular transport in pollen germination and organelle dynamics. This assertion is further supported by the irreversible growth cessation upon transfer to a fresh medium, emphasizing the irreplaceable role of autophagy (Fig. 3; 1 S). The selective impact of BFA on pollen tube elongation serves as a poignant reminder of the multi-dimensional involvement of autophagy across distinct germination phases (Fig. 4). The cascading effects of autophagy inhibition on organelle dynamics within germinating pollen grains become evident in the presence of autophagy-disrupting agents. The discernible deviations in the Golgi apparatus and amyloplasts underscore a regulatory intersection where autophagy and organelle remodeling intersect.

In addition to its effects on amyloplasts and other organelles, our observations, employing BODIPY 493/503 staining, have unveiled the critical role of autophagy in regulating lipid body dynamics during pollen germination (Fig. 3). Under normal conditions, we have observed a dynamic process wherein lipid bodies, essential for pollen tube growth and elongation, exhibited a distinct redistribution pattern. However, the presence of autophagy inhibitions notable disrupted these vital lipid body dynamics. In the absence of autophagy, the typical polarization of lipid body distribution toward the pollen tube tip, a process crucial for polarized growth, was absent. This observation underscores the close connection between autophagy activity and the efficient mobilization of lipid bodies from the pollen grain to the pollen tube. Autophagy disruption not only affects the differentiation of organelles like amyloplasts but also significantly perturbs the normal distribution of lipid bodies. This disruption likely contributes to the observed growth impediments and morphological deviations during germination (Phillips et al. 2008). The molecular cascades and regulatory nodes engaged by autophagy during germination require thorough exploration.

Moreover, the presence of vacuoles within pollen grains has long been acknowledged, yet their precise origins, developmental trajectory, and functional implications during pollen maturation remain enigmatic. Although a unified theory of vacuole ontogeny in plants is lacking, it is noteworthy that autophagy is elevated in growing pollen tubes and is required for pollen tube growth (Yan et al. 2023). Two distinct mechanisms for the formation of autophagosome-like compartments have been proposed, differing in the origin of the encircling membrane. The first involves the fusion of tubular provacuoles originating from the trans-Golgi network (TGN), encapsulating portions of the cytoplasm, subsequently leading to membrane degradation and enclosed cytoplasm. This progression culminates in the formation of autolysosomes, and further fusion of smaller autolysosomes results in the maturation of a vacuole. In contrast, the second mechanism sacs emerge from the smooth endoplasmic reticulum (ER) that envelop the cytoplasm, fusing among themselves, leading to inward expansion and eventual vacuole formation (Fig. 7).

Within germinating lily pollen, the autophagic machinery governing the degradation of stored reserves seems to align more closely with the latter mechanism. Substantiated by the observation that brefeldin A, a substance disrupting the default secretory pathway from the TGN, does not hinder the development of tubular ER-enveloped amyloplasts and lipid bodies during pollen germination, rigorous ultrastructural examination suggests the involvement of two distinct autophagic pathways in the mobilization and utilization of amyloplasts and lipid bodies during germination (Fig. 7). Lipid bodies, crucial for pollen tube growth, exhibit intriguing behavior in the presence of BFA. They are frequently enclosed within a double-layered membrane, reminiscent of autophagosome-like structures. This finding suggests that BFA disrupts the normal dynamics of lipid bodies and their mobilization from the pollen grain to the growing pollen tube. The entrapping of lipid bodies within autophagosome-like structures signifies their involvement in autophagy processes. The fate of newly synthesized amyloplasts remains ambiguous; however, the envelopment of nascent amyloplasts, cytoplasmic segments, and organelles within tubular ER-formed autophagosomes, implies their potential transformation into autolysosomes, ultimately contributing to vacuole formation. This proposition gains support from the co-localization of ER chaperone (BiP), α-amylase, and papain-type proteinase (SH-EP) within amyloplasts (Fig. 5). Conversely, autophagosomes harboring lipid bodies arising from tubular ER are anticipated to merge with pre-existing vacuoles, transferring their cargo for degradation within the vacuolar lumen. Additionally, our findings from the immunogold labeling of ATG8 reveal its presence in both starch granules and lipid bodies (Fig. 6). This dual localization further underscores the involvement of ATG8 in the regulation of autophagic processes related to the dynamic interplay between amyloplasts, lipid bodies, and vacuoles during lily pollen germination.

The intricate interplay between autophagy disruption and lily pollen germination accentuates the delicate equilibrium governing cellular processes and developmental outcomes. Autophagy disruption impedes germination and compromises the integrity of crucial organelles like amyloplasts and lipid bodies. The underlying molecular cascades and regulatory nodes engaged by autophagy during germination warrant meticulous examination.

During pollen germination, proplastids transform into amyloplasts, driven by carbon sources synthesizing starch granules. The ER, involving NSF and PI3 kinase, is crucial in this process, orchestrating proplastid-to-amyloplast transformation. ER is also vital in forming autophagosomes, enclosing lipid bodies, and forming double-membrane autophagosomes. This ER involvement complicates pollen development regulation (Singh et al. 2021). Reserves stored in starch granules and lipid bodies are degraded through autolysosome formation, ensuring efficient resource utilization for pollen tube growth. Fusion events between lipid bodies and autolysosomes culminate in the formation of vacuoles, promoting pollen tube growth and marking a transition from reserves to active growth. This dynamic process underscores the intricate interactions among organelles in the context of reproductive success **(**Fig. 7**)**.

In summary, the interplay between autophagy and lily pollen germination provides valuable insights into cellular processes in plant development (Su et al. 2020). Autophagy’s role in maintaining cellular equilibrium and its involvement in germination underscores its significance in plant reproductive strategies (Minina et al. 2013). This complex interplay deepens our understanding of plant growth and sheds light on the dynamic orchestration governing developmental events.

## Materials and methods

### Plant materials

Mature pollen grains of Easter lily (*Lilium longiflorum* Thunb. cv Avita) were obtained from anthers 1 day after anthesis. These pollen grains were desiccated by air-drying on a bench for 2 days following collection. The Easter lily bulbs were procured from a local farm, Foreport Enterprises Co., Ltd, Taipei, Taiwan. The bulbs were cultivated in a greenhouse under ambient conditions at Academia Sinica.

### Pollen germination and osmotic pressure treatment

In the experimental procedure, lily pollen grains were subjected to germination in a culture medium. Approximately 10 g of lily pollen were subjected to in vitro germination for a duration of 90 min. The germination was conducted in a medium with the following composition: 1.27 mM CaCl_2_, 0.162 mM H_3_BO_3_, 0.99 mM KNO_3_, and 290 mM sucrose, adjusted to a pH of 5.2. Two variations of the germination medium were employed: one with 290 mM sucrose and the other with the addition of 290 mM pentaerythritol. The pentaerythritol-enriched medium was utilized for pollen observation at time intervals of 30, 60, and 90 min during the germination process.

### Pollen germination and autophagy inhibitor treatment

The pollen grains were subjected to germination using the aforementioned germination solution. Subsequently, the pollen tubes were allowed to germinate in the presence of autophagy inhibitors. Specifically, 3-methyladenine (3-MA) was employed for a duration of 1 h, N-ethylmaleimide (NEM) for 15 min, and brefeldin A (BFA) for a range of 1 to 2 h. The effects of these treatments, including lipid body distribution, were observed using FLUOVIEW FV3000 confocal fluorescence microscopy. The length of the pollen tubes was measured at hourly intervals during this treatment period.

### Tissue preparation and immunolocalization for transmission electron microscopy

For ultrastructural observation, the desiccated pollen grains were washed from anthers and subsequently fixed in a solution comprising 2.5% glutaraldehyde and 4% paraformaldehyde in 100 mM phosphate buffer (pH 7.0) for 4 hours at room temperature. The fixed pollen grains were then rinsed three times in the same buffer for 15 minutes each time. The samples were post-fixed using 1% OsO_4_ in the buffer, followed by a rinse in the buffer as previously described. Dehydration was carried out using a graded series of acetone, and the samples were subsequently embedded in Spurr’s resin. Thin sections of 70 nm were cut using a Leica Reichert Ultracut S microtome and collected on nickel grids. These sections were post-stained with 6% uranyl acetate and 0.4% lead citrate, rinsed, and examined using a Phillips CM100 electron microscope based in Eindhoven, The Netherlands. In the rapid freeze preparation, the washed pollen grains were rapidly frozen using liquid nitrogen slush. Anhydrous methanol was used for freeze substitution in Leica EM AFS (automatic freeze substitution). The samples were maintained at -85°C for 2 days and then − 25°C for 1 day. Infiltration with LR Gold, including the addition of 0.1% benzil, was carried out at -25°C for 3 days. Polymerization was achieved using UV light, with one day at -25°C and two days at 25°C. Ultrathin sections of 100-120nm were cut using a Reichert Ultracut S microtome (Leica, Vienna, Austria) and collected on nickel grids of either 50 or 100 mesh. Samples intended for immunolocalization were fixed in a solution containing 0.1% glutaraldehyde and 4% paraformaldehyde in 100 mM phosphate buffer (pH 7.0) for 4 hours at room temperature. After fixation, the samples were rinsed three times for 15 minutes each in the buffer and dehydrated through a series of ethanol concentrations. The samples were then embedded in LR White resin. Double immunolabeling with two distinct rabbit polyclonal antibodies was performed following a modified approach based of Jauh *et al*. (Jauh, 1999). Thin sections on grids were subjected to blocking with PBS containing 0.05% Tween 20, 0.2% gelatin, 0.5% BSA (PBST), and 3% normal donkey serum at room temperature for 30 minutes. The grids were incubated with the first set of primary polyclonal antibodies against *Arabidopsis* COP8 (AtCOP8, Affiniti Research, Exeter, UK), diluted 1:100 in PBST at room temperature for 1 hour. Subsequently, the grids were rinsed five times with ddH_2_O and incubated with goat anti-rabbit F(ab’)_2_ fragments conjugated to 10 nm gold particles (British BioCell International, Cardiff, UK) in 1:2 dilution at room temperature for 1.5 h. After another round of rinsing, the grids were incubated with the second set of primary polyclonal antibodies (anti-Wx proteins) at a dilution of 1:25 for 2 h at room temperature. Control grids were subjected to incubation with the blocking buffer only. After washing the grids multiple times, a 1:25 dilution of donkey anti-rabbit IgG (H + L) antiserum conjugated to 18 nm gold particles (Jackson Immuno Research, West Grove, PA, USA) was applied to the grids at room temperature for 30 min. Following additional rinses, the grids were post-stained with uranyl acetate and lead citrate, rinsed again and examined under an electron microscope.

### Electronic supplementary material

Below is the link to the electronic supplementary material.

Supplementary Material 1

## Data Availability

Data sharing is not applicable to this study as no new data were created or analyzed.
