# Supplementary figures and images for: Dynamic organelle changes and autophagic processes in lily pollen germination
Source: Bot Stud. 2024 Jan 26;65:5. doi: 10.1186/s40529-024-00410-6 (PMC10811312; doi:10.1186/s40529-024-00410-6)

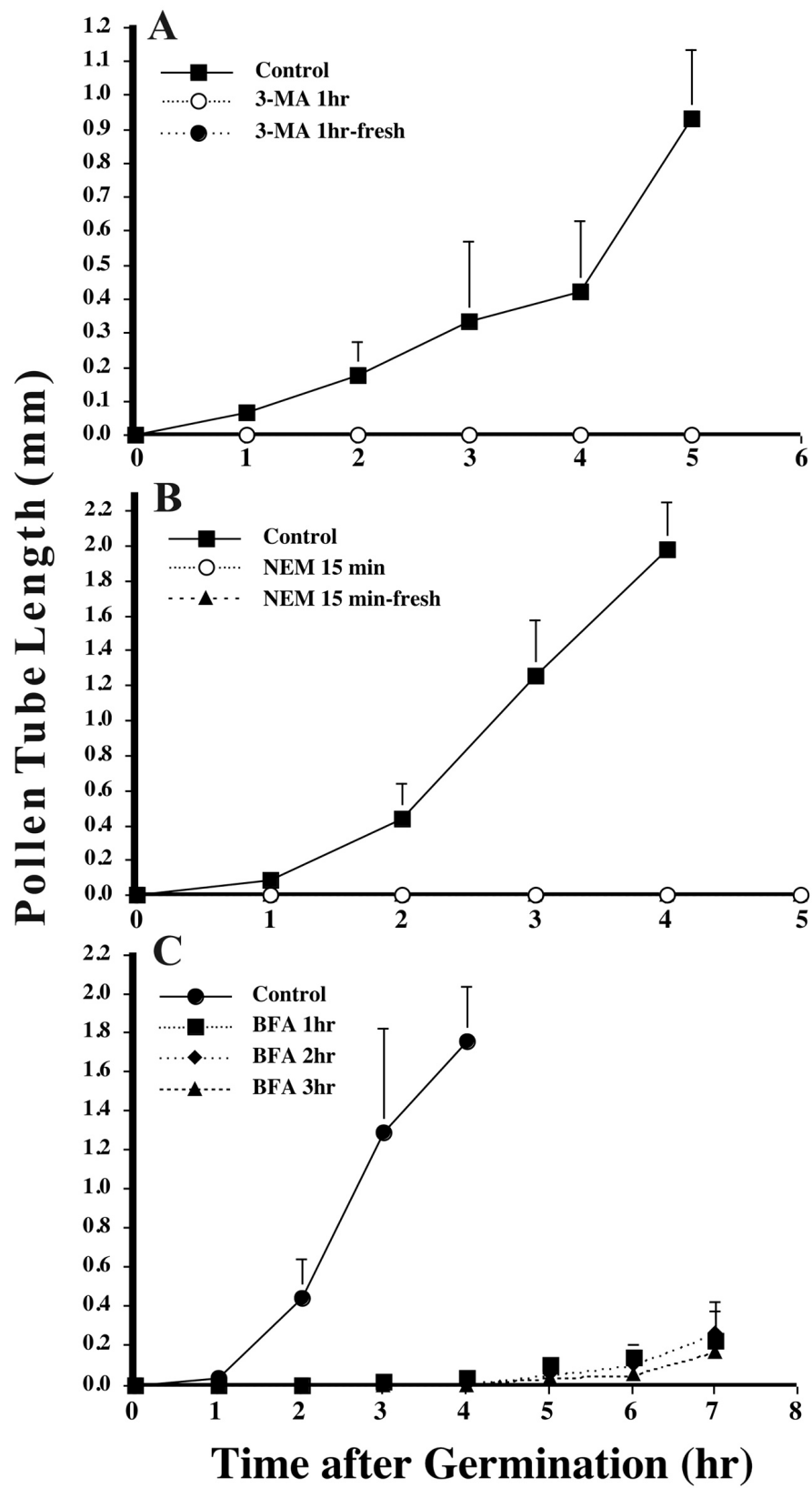

Supplement: Supplementary file 1 — Supplementary Material 1 [file 40529_2024_410_MOESM1_ESM.pdf]
